# Physicochemical and cytotoxicity analysis of green synthesis carbon dots for cell imaging

**DOI:** 10.17179/excli2019-1465

**Published:** 2019-06-27

**Authors:** Zahra Fatahi, Neda Esfandiari, Hamide Ehtesabi, Zeinab Bagheri, Hossein Tavana, Zahra Ranjbar, Hamid Latifi

**Affiliations:** 1 Protein Research Center, Shahid Beheshti University G.C., Tehran, Iran; 2 Department of Bioengineering and Bionanotechnology, Faculty of Life Sciences and Biotechnology, Shahid Beheshti University G.C., Tehran, Iran; 3 Department of Biomedical Engineering, The University of Akron, Akron, OH 44236, USA; 4 Institute for Color Science and Technology (ICST), Department of Surface Coatings and Corrosion, Tehran, Iran; 5 Laser & Plasma Research Institute, Shahid Beheshti University G.C., Tehran, Iran

**Keywords:** carbon dots, bitter orange, imaging, cytotoxicity

## Abstract

Carbon dots (CDs) have outstanding optical properties, biocompatibility, and photostability, making them attractive for imaging applications. A facile and green one-step hydrothermal synthesis method is proposed, which can be safely used in a wide range of applications such as chemical sensing, bioimaging, and optoelectronics. In this study, we report green synthesis of carbon dots from bitter orange juice (Citrus Aurantium) by hydrothermal treatment for the first time. We studied effects of time, temperature, and pH on fluorescence of CDs, characterized them using various spectroscopic and microscopic methods, and evaluated their toxicity to different cell lines. Identifying an optimum reaction condition of 180 ºC for 7 h heating gave CDs that showed pH-dependent fluorescence, with the largest fluorescence at a pH of 7.0. The CDs were 1-2 nm in size with a spherical morphology and negative surface charge. The CDs showed a high quantum yield of 19.9 %, reasonable photostability, excellent water solubility, and long fluorescence lifetime. A one step hydrothermal rout led to various hydrophilic functional groups on the surface of the CDs. Our results showed that the CDs were non-toxic over a large concentration range and effective for imaging of cells, indicating their potential as imaging probes in medical diagnostics and biosensor applications.

## Introduction

Carbon dots (CDs) are a recently-discovered biocompatible class of fluorescent carbon nanoparticle family, with a typical size of less than 10 nm (Jelinek, 2017[20]; Gyulai et al., 2019[18]). In recent years, these light-emitting quantum-sized carbon dots have gained attention due to their small size, chemical inertness, high water solubility, lack of optical blinking, strong photo-stability (Qu et al., 2012[33]; Guo et al., 2013[17]), tunable emission wavelength (Shen et al., 2013[40]), electroluminescence characteristics, low cytotoxicity, and excellent biocompatibility (Chatzimitakos et al., 2017[5]). Due to these benefits, CDs are promising agents for applications in analytical chemistry, bioimaging, pharmacy, drug delivery, optoelectronic devices, and biosensing (Mehta et al., 2015[28]; Atchudan et al., 2016[2]; Li et al., 2017[23]). Efforts have been made to synthesize CDs with high fluorescent intensity and different emission properties. In general, CDs are synthesized using either top-down or bottom-up methods. The top-down approaches are mainly performed by arc discharge (Xu et al., 2004[51]), laser ablation (Sun et al., 2006[43]) and electrochemical exfoliation (Ming et al., 2012[29]). Bottom-up approaches contain microwave-assisted carbonization (Wang et al., 2010[46]), pyrolysis (Shi et al., 2015[41]), hydrothermal mechanism and aqueous based methods (Suvarnaphaet et al., 2016[44]). In the top-down methods, graphite or multi-walled carbon nanotubes are crushed to obtain nanoscale carbon, whereas in the bottom-up methods, carbonization of carbohydrates or simple organic acids is used for the production of fluorescent CDs (Sahu et al., 2012[35]; Atchudan et al., 2016[2]; Li et al., 2018[22]). However, most of these synthesis methods are cumbersome and entail time-consuming processes, surface passivation treatments to improve their water solubility, high cost, and low quantum yield (Sahu et al., 2012[35]; Sharma et al., 2016[37]). According to previous studies, CDs obtained from candle soot, lampblack or graphene oxide by using heating under reflux in nitric acid, microwave-assisted heating under reflux, microwave-hydrothermal approaches have shown low quantum yield ranging from 1-3 % (Wang et al., 2011[48]). Currently, producing self-passivated carbon dots in one step is a challenging task (Sahu et al., 2012[35]; Sharma et al., 2017[38]).

New methods have been developed to use different natural materials such as fruit juice (Mehta et al., 2015[28]; Atchudan et al., 2016[2]; Ding et al., 2017[8]), vegetable extract (Wang et al., 2014[47]), and animal-derived and waste materials (Sharma et al., 2017[38]) as a source of carbon to prepare carbon dots nanoparticles. Green chemistry has brought considerable benefits of using cost-effective bio-waste materials, reducing toxic chemicals, simplifying the synthesis, and omitting post-treatment processes (Ensafi et al., 2017[14]). Among all bottom-up approaches, a hydrothermal method is considered as a one-step, simple, environmentally friendly, and controllable approach to produce amphibious CDs in aqueous solution with high quantum yield, without a need for any post-treatments (Shen et al., 2017[39]).

We used a hydrothermal method to synthesize highly fluorescent carbon dots from bitter orange juice for the first time. This green precursor contains sucrose, glucose, fructose, citric acid, and ascorbic acid, and provides a major carbon source for the synthesis. Moreover, these components are composed of H and O elements, facilitating the bonding of functional groups with the CDs surface. Consequently, water-solubility and fluorescent property of CDs increases due to these functional groups (Sahu et al., 2012[35]; Shen et al., 2017[39]). We synthesized CDs at different temperatures and time periods and studied their optical, morphological, and biocompatibility properties. To investigate CDs potential for bioimaging, we evaluated the synthesized green CDs' entrance to the cells and carried out a cytotoxicity assay to confirm that these nanoparticles are biocompatible and safe to cells.

## Materials and Methods

### Materials

Bitter oranges were purchased from a local market. RPMI medium, fetal bovine serum (FBS), penicillin/streptomycin, trypsin/EDTA, and phosphate buffered saline (PBS) were obtained from Gibco. MTT [3- (4,5-Dimethylthiazol-2-yl)-2,5-Diphenyltetrazolium Bromide] was acquired from Sigma and lactate dehydrogenase cytotoxicity assay kit was purchased from Promega. Ultrapure water was used in this experiment of this study.

### Preparation of CDs

Bitter oranges were used as the carbon source to synthesize CDs. Briefly, fresh bitter oranges were rinsed with ultrapure water and dried with a paper towel. Then, they were put into fruit juicer to obtain bitter orange juice. The measured amount of juice was transferred into 50 ml Teflon-lined stainless steel autoclave and was heated at 120 ºC for different time periods of 2.5 h and 7 h. Synthesis of CDs was performed at temperature 180 ºC for 7 h. After hydrothermal-carbonization, the autoclave was cooled down to room temperature. The resulting dark brown solutions were centrifuged at 10,000 rpm for 15 min to remove large particles and the brown supernatant was collected. Finally, the synthesized CDs solution were passed through a 0.22 µm filter membrane to omit larger particles and impurities. The CDs solution was diluted with ultrapure water to obtain a 5 mg/ml solution that was stored in dark at 4 ºC for further analysis. 

### Quantum yield measurements

Quantum yield (QY) of CDs was determined by comparative method using quinine sulfate (0.1 M H_2_SO_4_ QY = 0.54 at 365 nm excitation) as a standard reference.


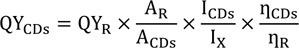


Here, QY is the quantum yield, I indicates fluorescent emission intensity, A represents the absorbance measured at the excited wavelength, and η represents the refractive index of the solvent. The subscript R connotes the known reference fluorescent (Hu et al., 2009[19]; Ding et al., 2017[10]).

### Characterization of CDs

Analysis of optical properties of CDs was performed using PerkinElmer LS 45 fluorescence spectrophotometer. UV-Vis absorption spectra were recorded on PerkinElmer lamda 2 UV-visible spectrophotometer. The identification of functional groups was done using a Bomem FT-IR. Carbon, hydrogen, and nitrogen contents were determined by a Vario EL III elementar instrument (Elmentar Analysensysteme GmbH). Horiba SZ-100 zeta potential analyzer was used for zeta potential measurements. Particle size distribution of CDs was characterized by DLS (Nanophox, Sympatec GmbH). The shape and surface morphology of CDs nanoparticles were evaluated by atomic force microscopy (AFM). Of the CDs solution, 1 μL was deposited onto a freshly cleaved mica surface and dried in air. The AFM instrument was used to capture the morphology of CDs. AFM imaging of samples was conducted on a DME instrument (Dualscope C-26, with DME-SPM v2.1.1.2 software) by tapping-mode in air under ambient conditions.

### Stability of CDs 

To investigate CDs stability, fluorescent intensity was evaluated by PerkinElmer LS 45 fluorescence spectrophotometer 8 months after synthesis. Also, CDs solutions with different pH values were prepared using 0.5 M NaOH. For adjusting the pH to 5, 7, 9, and 11, different amounts of 0.5 M NaOH was added to CDs solution. After 30 minutes, fluorescent intensity measurement was performed at room temperature.

### Cell culture, viability, and toxicity analysis

SKBR3 breast adenocarcinoma cell line and NIH/3T3 mouse embryonic fibroblast were seeded in 24-well plate using RPMI-1640 (Roswell Park Memorial Institute) containing 10 % (v/v) FBS, 50 U/ml penicillin, and 50 µg/ml streptomycin. The cells were grown in a 5 % CO_2_ incubator at 37 °C. Cellular viability was measured by the activity of mitochondrial enzyme in live cells. Briefly, SKBR3 and NIH 3T3 cells were seeded into 96-well tissue culture plates at a density of 1×10^4^ cells per well in RPMI-1640 containing 10 % FBS and 1 % penicillin-streptomycin. After 24 h, the culture medium was replaced with medium containing CDs at various concentrations of 10, 20, 40, 80, 160, 320, 640, 1280 µg ml^-1^. Cells in the presence of CDs were incubated for 24 h and 48 h, whereas cells without treatment with CDs were used as a negative control. For analysis, 20 µl of 3-[4,5-dimethylthiazol-2-yl]-2,5-diphenyltetrazolium bromide (MTT solution, 5 mg ml^-1^) was added to each well. The plate was placed in an incubator at 37 ºC for 4 h. Then, the MTT solution was removed and 100 µL of DMSO solution was added to dissolve the formazan compound. After 15 minutes, incubation at ambient temperature, the absorbance was measured at 570 nm with a microplate reader (BioTek, ELx 800). The experiments were performed in triplicate and repeated three times.

Cell membrane integrity was evaluated using LDH assay. After 45 min of incubating cells with 10 - 1280 µg ml^-1^ filtered sterilized CDs. Lysis buffer solution was added to control wells for 45 minutes as a positive control (MR). The supernatant was aspirated from each well and transferred into a 96-well plate. The absorbance of the supernatants was recorded at 490 nm using a microplate reader (ELx 800, BioTek). The experiments were independently repeated in triplicate.

### Imaging of cellular uptake of CDs 

SKBR3 cells were grown in 24-well tissue culture plates at a density of 6×10^4^ cells per well. Then, 20 µg ml^-1 ^of filtered sterilized CDs in complete growth medium was added to the plates. The plates were incubated for 5 h (Liu et al., 2012[26]; Esfandiari, 2018[15]). After washing with BPS twice, the cells were imaged at 365 nm excitation and 470 nm emission using an Olympus inverted microscope. Cells without any treatment with CDs were considered as negative control.

### Statistical analysis

The data were analysed using analysis of variance (ANOVA) followed by LSD post hoc test, in SPSS 16.0. Results were reported as mean ± SEM. Before performing the level of significance for the tests, normality distribution was tested by Kolmogorov-Smirnov and Shapiro-Wilk tests. Significance was defined as p<0.001. All experiments were performed in triplicate and repeated three times. 

## Results and Discussion

**Figure 1:**
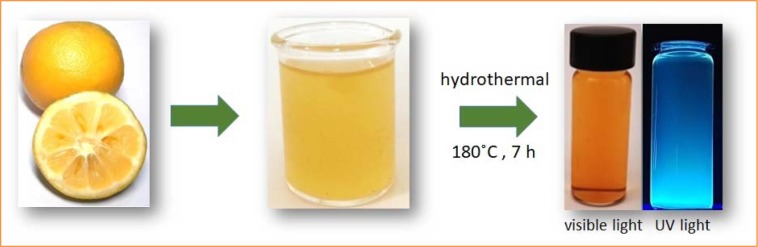
Schematic illustration of preparation of CDs from hydrothermal treatment of orange juice

Citrus Aurantium (bitter orange) extract is widely utilized in weight management products due to its five adrenergic amines: synephrine, *N*-methyltyramine, hordenine, octopamine, and tyramine which contribute to various metabolic processes by catecholamines and lypolysis (Sale et al., 2006[36]). Furthermore, it is used in sports performance products to improve stamina. Previous studies have not reported any adverse effect of bitter orange (Sale et al., 2006[36]; Stohs et al., 2012[42]). Among citrus varieties, bitter orange species have high acid and sugar contents that provide a considerable amount of carbon and hydrogen elements (Moufida and Marzouk, 2003[30]). For the first time in this study, we report an inexpensive green source for the synthesis of non-toxic CDs simply using hydrothermal carbonization of bitter orange fruit juice (Figure 1(Fig. 1)). The hydrothermal treatment along with decomposition of carbon source are precursors to produce the CDs. The citric acid content of bitter orange makes it a suitable source to produce CDs. We optimized synthesis conditions such as reaction time, reaction temperature, and pH to gain highly fluorescent CDs, as described below. 

### Optimization of CDs preparation 

**Figure 2:**
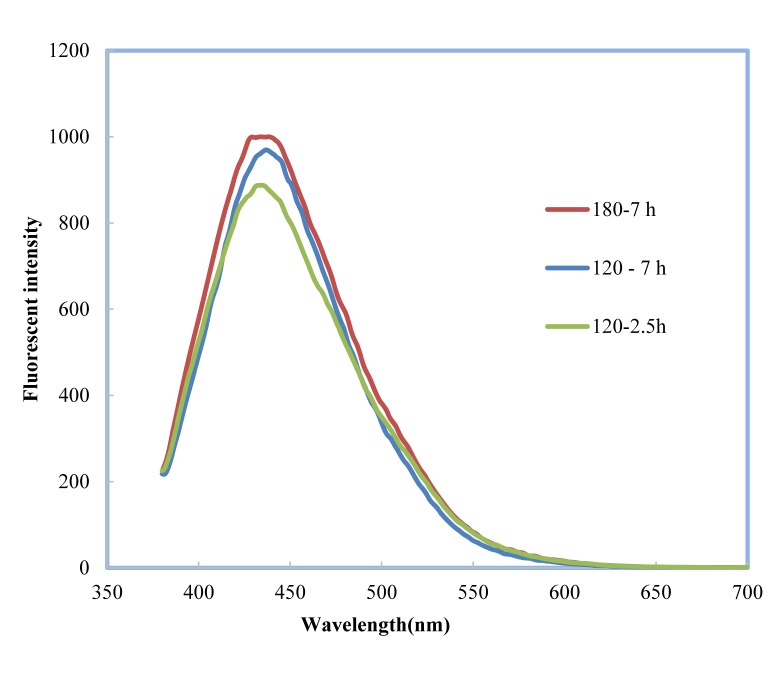
Fluorescent intensity of green synthesis CDs at different temperatures and time. CDs synthesized by hydrothermal treatment at 180 °C showed maximum fluorescent intensity.

Synthesis conditions have major effects on optical properties of CDs. We first studied effects of reaction time and temperature of CDs preparation. As shown in Figure 2(Fig. 2), the strongest fluorescent intensity was observed when bitter orange juice was heated at 180 °C for 7 h under ultraviolet illumination at 365 nm excitation that validated the formation of fluorescent CDs. High temperature and high pressure within the stainless steel autoclave resulted in dehydration and pyrolysis of bitter orange juice and synthesis of CDs. Higher temperature and longer time increased the carbonization degree and resulted in larger fluorescent intensities (Tyagi et al., 2016[45]; Li et al., 2017[22]). To confirm this finding, we measured the QY of the CDs using quinine sulfate as the reference. We obtained excellent CDs and the highest QY at high temperatures and long incubation time, but according to previous studies further increase in temperature beyond 180 °C led to fluorescent reduction (Yin et al., 2013[52]).

### Characterization of CDs

**Figure 3:**
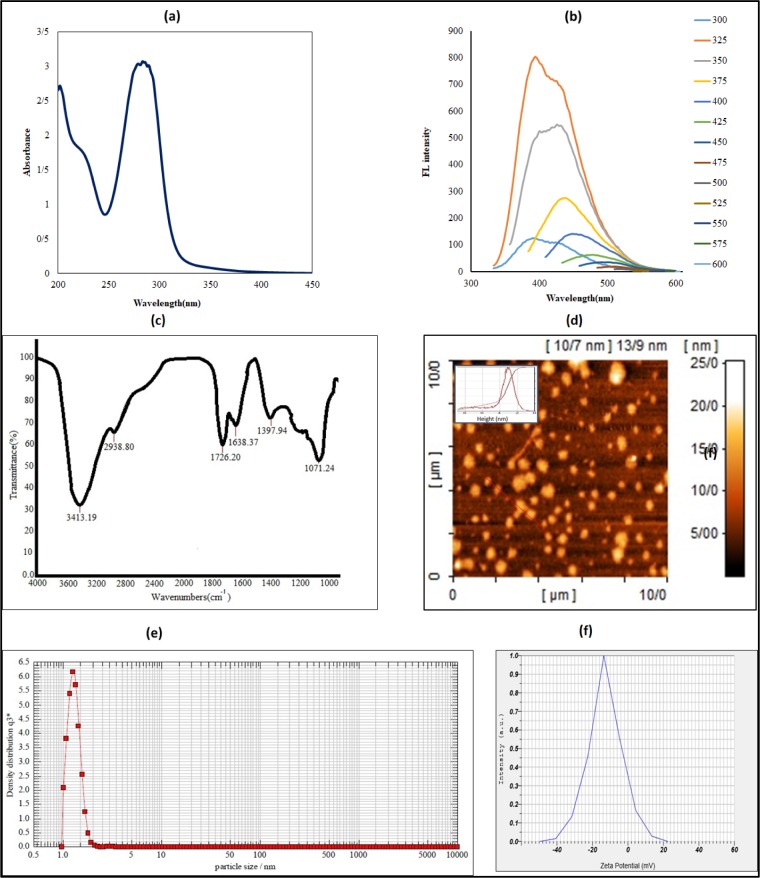
CDs characterization by UV-vis, fluorescent and Fourier transform infrared spectroscopy, atomic force microscopy, dynamic light scattering and zeta potential analyzer: (a) UV-vis absorption, fluorescence excitation (λ_ex_= 365 nm) spectra of CDs at 200-450 nm, (b) Fluorescence intensity of CDs at different excitation wavelengths

To obtain organic CDs from bitter orange, we carried out hydrothermal carbonization at 180 ºC. As shown in Figure 1(Fig. 1), hydrothermal treatment of bitter orange juice gave a dark brown solution, which implies carbonization of CDs. The CDs solution under UV light emitted a bright blue fluorescent light. The UV-visible spectrum of CDs showed a broad absorbance band at 280 nm (Figure 3a(Fig. 3)) corresponding to n-π* and π-π* transition of the C=O bonds of carboxyl group and conjugated C=C bonds (Ding et al., 2014[9]; Dubey et al., 2014[12]). Figure 3b(Fig. 3) shows the fluorescent spectrum emission of the CDs at different excitation wavelengths from 300 nm to 600 nm. The emission intensity increased from 300 nm to 325 nm, then gradually decreased up to 600 nm. This excitation-dependent fluorescent behavior indicates that the resulting CDs depend on sizes and fluorescent colors of nanoparticles. This phenomenon could also result from presence of surface states. These CDs possess various functional groups, which will be presented in the following. These functional groups result in a series of emissive traps between π and π* states. When a defined excitation wavelength illuminates fluorescence property of the CDs, a surface energy trap dominates the emission. While the excitation wavelength alters, another corresponding surface state emissive trap will become dominant. Hence the photoluminescence (PL) mechanism is controlled by both size effect and surface defect which is formed by surface oxidation. Surface defect can prepare excitons center as a consequence, fluorescence will be increased (Ding et al., 2016[11]). The finding is consistent with previous studies (Sahu et al., 2012[35]; Tyagi et al., 2016[45]; Ensafi et al., 2018[13]). Figure 3c(Fig. 3) shows FT-IR spectra of CDs. The absorption band at 3413.19 cm^-1^ corresponds to the stretching vibrations of O-H and N-H. The absorption bands at 1726, 1397, and 1071.24 cm^-1^ correspond to stretching vibrations of C-H, C-H_3_, and C-O groups. Moreover, C=O stretching vibration at 1738.37 is considered as a specific feature of CDs (Zhang et al., 2010[53]; Chandra et al., 2012[4]). These bonds confirm that hydroxyl, epoxy, carbonyl, carboxylic acid, and aminopropyl groups formed on the surface of the CDs and making them water soluble (Sahu et al., 2012[35]; Gao et al., 2015[16]). Elemental analysis indicated that CDs contain 37 % carbon, 5.8 % hydrogen, and 0.5 % nitrogen (Table 1(Tab. 1)).

Figure 3d(Fig. 3) shows a typical AFM image that presents morphology of CDs. Furthermore, it shows a height distribution of about 2-4 nm corresponding to 3-5 layers of graphite (Pan et al., 2010[32]; Li et al., 2017[24]). In this image, CDs appear to be different in size, which may result from carbon dots aggregation also observed in other studies (Dinç, 2016[7]). DLS results indicated that the mean diameter of CDs was 1-2 nm. The CDs were monodispersed (Figure 3e(Fig. 3)), hydrophilic, and very small, allowing them to cross membrane of living cells. These results were consistent with previous reports (Mehta et al., 2017[27]; Wei et al., 2017[49]). Furthermore, zeta potential measurements showed that the CDs were negatively charged (-12.8 mV) (Figure 3f(Fig. 3)), which implies formation of hydroxyl and carboxylate groups on the surface of the CDs as reported before (Bandi et al., 2018[3]).

**Figure 4:**
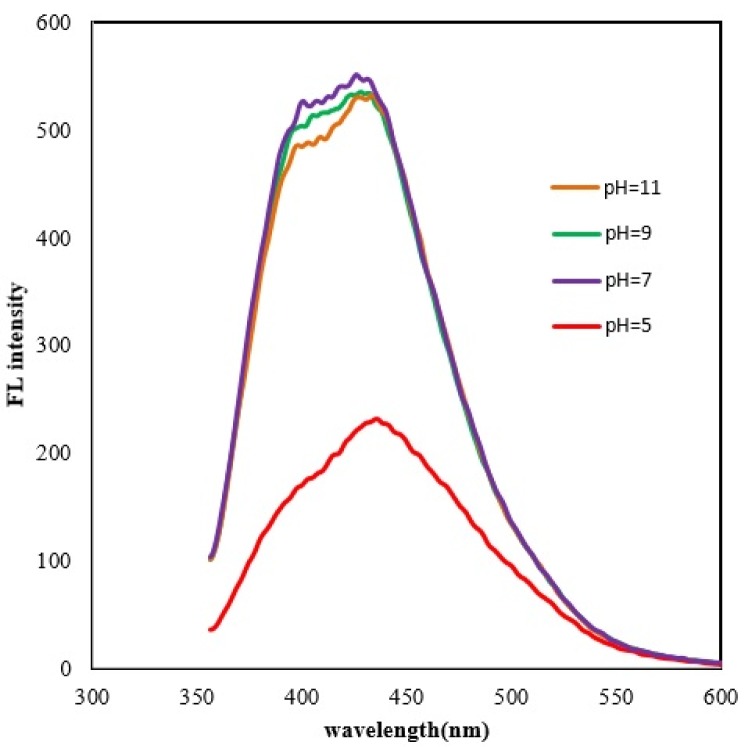
pH effect on the fluorescence of CDs. Optimum Fluorescence intensity was measured at neutral pH of 7.0

To investigate the influence of pH, we used CDs solutions of different pH values (Figure 4(Fig. 4)). The fluorescent intensity of CDs increased from a pH of 5 to 7 and then remained fairly steady with very small decreases from a pH of 7 to 9 and to 11. The maximum fluorescence intensity was observed at a pH of 7. Other studies also reported the strongest photoluminescence intensity in a neutral pH. Moreover, fluorescence decreased in both acid and alkaline conditions (Alam et al., 2015[1]; Kumar et al., 2018[21]), although increase in fluorescent intensity with increase in pH has also been reported (Zhong et al., 2016[54]). 

### Stability of CDs and their QY

To evaluate the long-term stability of CDs, fluorescent intensity of an aqueous solution of CDs was measured after 8 months. Interestingly, CDs completely retained their fluorescent property. Photo-stability studies suggest that CDs have a great potential in biological applications such as cell imaging, biosensing, and drug/gene delivery (Bandi et al., 2018[3]). We note that the CDs solution could also be dried in a freeze dryer to a powder.

Our measurements showed that the fluorescence QY of CDs at 365 nm excitation was about 19.90 %. The high quantum yield is due to the self-surface passivation of CDs with nitrogen (N) content group, which can effectively improve optical property of CDs. Functionalization (N) of CDs by various elements, notably nitrogen, can lead to radiative electrons recombination stand holes and result in high QY (Bandi et al., 2018[3]). The nitrogen atoms transfer electron into CDs and alter the internal electronic environment, which cause an increase in the fluorescent property (Xu et al., 2016[50]). Several reports have shown that green CDs produced from natural precursors have QY approximately ranging from 1 % to 30 % (Sharma et al., 2017[38]). Therefore, CDs derived from bitter orange are among those with a high QY. 

### CDs as imaging agents and toxicity to cells

**Figure 5:**
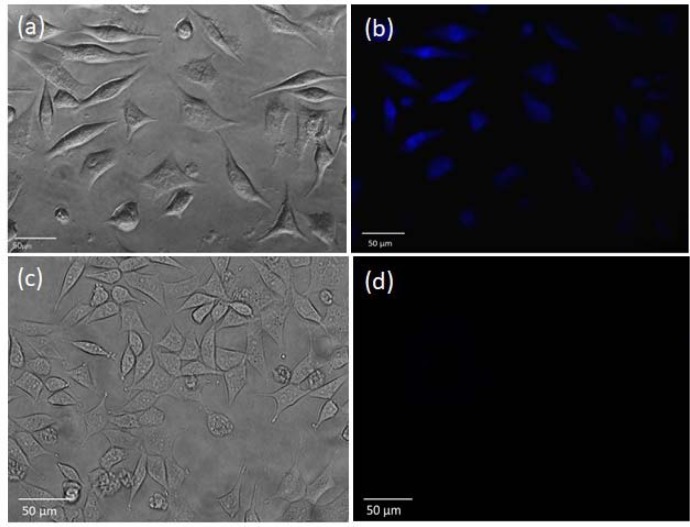
Phase and fluorescence images of SKBR3 cells incubated with (a-b) 80 μg/mL of the CDs for 5 h, and (c-d) without CDs (negative control)

We used SKBR3 breast cancer cells to explore the potential of CDs as a bioimaging agent. A concentration of 20 µg/ml was used as a low concentration of CDs. *D*istinct blue fluorescent color was emitted from the treated cells, indicating that CDs crossed the membrane of the cells (Figure 5a-b(Fig. 5)). On the other hand, the fluorescence emission was absent in the control cells (Figure 5c-d(Fig. 5)). As shown in phase images, cells retained their normal morphology after treatments, indicating the biocompatibility of the CDs (Sachdev and Gopinath 2015[34]). It has been reported that due to their small size, CDs cross the cell membrane through endocytosis. Consequently, they can also penetrate into the cell nucleus (Sharma et al., 2017[38]). Hence, the CDs from bitter orange can be used as a fluorescence probe.

**Figure 6:**
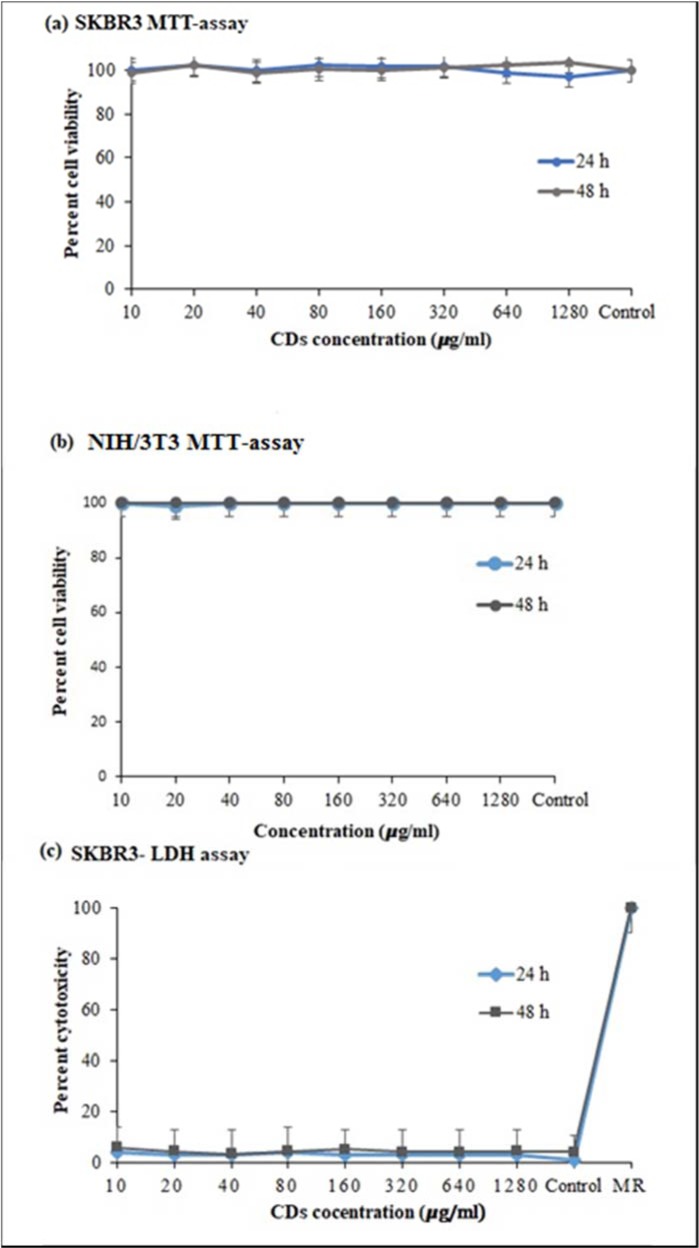
Cytotoxicity tests. (a) MTT assay of SKBR3 cancer cells, (b) MTT assay of NIH/3T3 normal cells (after 24 h incubation of CDs), (c) LDH assay of SKBR3 cell line. The graph shows no obvious toxicity of CDs to both cancer and normal cells even at high concentrations. Values represent mean ± SEM (P ≤ 0.001, one way ANOVA).

Biocompatibility and low cytotoxicity of CDs are critical in biomedical applications. Therefore, we evaluated the cytotoxicity of CDs using MTT colorimetric assay and LDH assay. We treated both SKBR3 and NIH3T3 cells with different concentrations of CDs to determine the cytotoxicity of CDs. As shown in the result from MTT assay (Figure 6a(Fig. 6)), SKBR3 cell viability was not significantly changed with increasing concentrations of CDs between 10 to 1280 µg/ml and 24 h and 48 h of incubation. We obtained a similar result with the normal NIH/3T3 cells (Figure 6b(Fig. 6)). Thus, the green CDs were compatible with human cells and did not have any toxic effects. This is consistent with few other reports (Alam et al., 2015[1]; Sachdev and Gopinath 2015[34]; Li et al., 2017[25]; Bandi et al., 2018[3]). We also used LDH assay to study potential cell membrane damage due to CDs. The result in Figure 6c(Fig. 6) shows that after 24 h and 48 h of incubation of SKBR3 cells with the CDs, there was only a low LDH release of <~5 % regardless of concentration of CDs up to 1280 µg/ml compared to the negative control (without CDs). According to other articles this result is consistent (Nurunnabi et al., 2013[31]; Chong et al., 2014[6]). Taken together, these results show complete biocompatibility of the bitter orange-derived CDs.

## Conclusion

We introduced a facile, green, and large-scale synthesis of carbon nanoparticles from a low cost and available natural precursor, bitter orange, through a one-step hydrothermal method. The bitter orange-derived CDs had a small size, high quantum yield, were water soluble, and showed stable fluorescence. These nanoparticles emitted bright blue fluorescent under UV light. Cells treated with the CDs maintained a normal morphology and emitted a strong fluorescence. The CDs were totally biocompatible and non-toxic to both normal and cancer cell lines. These green CDs may be used in various applications such as imaging of live cells. 

## Figures and Tables

**Table 1:**
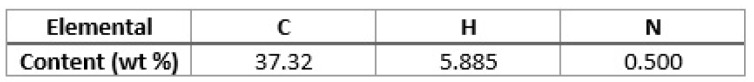
CHN elemental analysis of synthesized CDs
